# Metabolic Risks Are Increasing in Non-B Non-C Early-Stage Hepatocellular Carcinoma: A 10-Year Follow-Up Study

**DOI:** 10.3389/fonc.2022.816472

**Published:** 2022-02-02

**Authors:** Yen-Po Lin, Pei-Ming Wang, Ching-Hui Chuang, Chee-Chen Yong, Yueh-Wei Liu, Pao-Yuan Huang, Chih-Chien Yao, Ming-Chao Tsai

**Affiliations:** ^1^ School of Medicine, Chung-Shan Medical University, Taichung, Taiwan; ^2^ Department of Family Medicine, Kaohsiung Chang Gung Memorial Hospital, Kaohsiung, Taiwan; ^3^ Department of Nursing, Meiho University, Pingtung, Taiwan; ^4^ Liver Transplantation Center and Department of Surgery, Kaohsiung Chang Gung Memorial Hospital and Chang Gung University College of Medicine, Kaohsiung, Taiwan; ^5^ Division of Hepato-Gastroenterology, Department of Internal Medicine, Kaohsiung Chang Gung Memorial Hospital and Chang Gung University College of Medicine, Kaohsiung, Taiwan; ^6^ Graduate Institute of Clinical Medical Sciences, College of Medicine, Chang Gung University, Taoyuan, Taiwan

**Keywords:** NBNC, CHB, hepatocellular carcinoma, recurrence, metabolic associated fatty liver disease (MAFLD), metabolic dysfunction

## Abstract

**Background:**

Non-B, non-C hepatocellular carcinoma (NBNC-HCC) may be related to metabolic syndrome, and the incidence of this tumor type is increasing annually. The definition of metabolic-associated fatty liver disease (MAFLD) proposed in 2020 may help to more accuratelyassess the association between metabolic syndrome and NBNC-HCC. However, this new concept has not yet been applied in NBNC-HCC research. Therefore, this study aimed to compare the clinicopathological characteristics of patients with NBNC-HCC and CHB-HCC diagnosed between 2009-13 and 2014-18, focusing on metabolic risk factors and the new concept of MAFLD.

**Method:**

Patients with BCLC-0/A-HCC who received curative hepatectomy between January 2009 and December 2018 were retrospectively assessed; the associations between clinicopathological characteristics and clinical outcomes of NBNC-HCC and CHB-HCC were analyzed by multivariate analysis.

**Result:**

Compared to patients diagnosed in 2009-13, the frequency of metabolic disorders in NBNC-HCC was significantly higher in 2014-18 [DM (*p*=0.049), HTN (*p*=0.004), BMI (*p*=0.017) and MAFLD (*p*=0.003)]; there was no significant change in patients with CHB-HCC. Moreover, CHB-HCC was an independent risk factor for HCC recurrence (HR, 1.339; 95% CI, 1.010-1.775, *p=*0.043) and death (HR, 1.700; 95% CI, 1.017-2.842, *p*=0.043) compared to NBNC-HCC.

**Conclusions:**

Therisk of MAFLD, obesity, DM, and hypertension in patients with early-stage NBNC have significantly increased in recent years, thus metabolic syndrome should be monitored in this special population. Moreover, NBNC-HCC tend to had a better prognosis than CHB-HCC, probably due to their distinct clinicopathological features.

## Introduction

Hepatocellular carcinoma (HCC) is the sixth most common cancer in the world and the fourth in terms of cancer mortality (1). HCC is generally considered to be related to multiple risk factors, including hepatitis B virus (HBV) or hepatitis C virus (HCV) infection, alcoholism, and metabolic syndrome (2, 3). Approximately 50% of all cases of HCC worldwide are associated with HBV infection, with a further 25% associated with HCV (4–7). Although most cases of HCC are related to viral infection, a substantial population of patients with HCC (5-20%) are seronegative for markers of both HBV and HCV infection [non-B, non-C (NBNC) hepatitis]. Moreover, the incidence of NBNC-HCC is growing rapidly (8–12).

The major cause of HCC in Taiwan is HBV infection, followed by HCV infection; similar observations have been reported in many other Asian countries (13–15). Due to the introduction of an universal HBV vaccination program for newborns and infants, development of antiviral therapies for HBV and HCV infection, and changes in lifestyle, the incidence of virus-related HCC has decreased over the last decade. However, the number of patients with HCC with neither HBV nor HCV infection, also known as NBNC-HCC, has been increasing annually; NBNC-HCC currently accounts for 11% of all cases of HCC in Taiwan (16). This trend has also been observed in South Korea and Japan (17, 18), which are HCV-endemic countries. Therefore, NBNC-HCC is emerging as a significant subgroup of HCC in areas of East Asia, despite this region being endemic for chronic hepatitis B (CHB) and CHC and having a high incidence of viral-related HCC.

The background and molecular mechanisms of NBNC-HCC remains unclear. Nonalcoholic steatohepatitis (NASH) and metabolic syndrome are considered as risk factors for HCC, especially for NBNC-HCC (19–22). However, a previous study had identified high proportion rate of occult HBV infection (OBI) in NBNC-HCC (23), which implies the complex interaction between CHB-HCC and NBNC-HCC, especially in Taiwan, an endemic area of CHB. This suggests that patients with non-viral-related HCC in Taiwan may have underlying OBI as a likely cause of HCC. However, due to lifestyle changes and the increased consumption of westernized foods in Taiwan, the number of patients exhibiting fatty liver and metabolic syndrome has increased drastically (24, 25). Furthermore, the HBV vaccination program for infants was implemented in Taiwan for more than 30 years, which had been proven to lower the frequency of OBI. We believe that the interaction between CHB-HCC and NBNC-HCC might be changing over time.

In 2020, a panel of experts proposed a change of the terminology from NAFLD to metabolic-associated fatty liver disease (MAFLD) (26, 27). The diagnosis of MAFLD is based on the presence of liver steatosis in addition to overweight/obesity, the presence of type 2 diabetes mellitus (T2DM), or the presence of metabolic dysregulation with at least two risk features including increased waist circumference, pre-diabetes, hypertension, hypertriglyceridemia, or low serum high-density lipoprotein (HDL)-cholesterol levels (26, 27). Accordingly, MAFLD is more likely to be associated with metabolic dysregulation-related events than NAFLD. However, to date, no study has investigated the association between the new concept MAFLD and NBNC-HCC.

Therefore, in the present study, we aimed to compare the clinicopathological features and prognosis of patients with NBNC-HCC and patients with CHB. Furthermore, we compared the clinicopathological characteristics of patients during two five-year periods (2009-13) and (2014-18), with a focus on the metabolic risk factors and the new concept of MAFLD.

## Patients and Methods

### Study Design and Ethics

This study was designed as a multicenter cross-sectional retrospective study in Taiwan. The Institutional Review Board of Kaohsiung Chang Gung Memorial Hospital approved this study (IRB number: 201701632A3) with a waiver of the requirement for informed consent owing to the retrospective design of the study with minimal risk to the participants.

### Study Population

**Figure 1:**
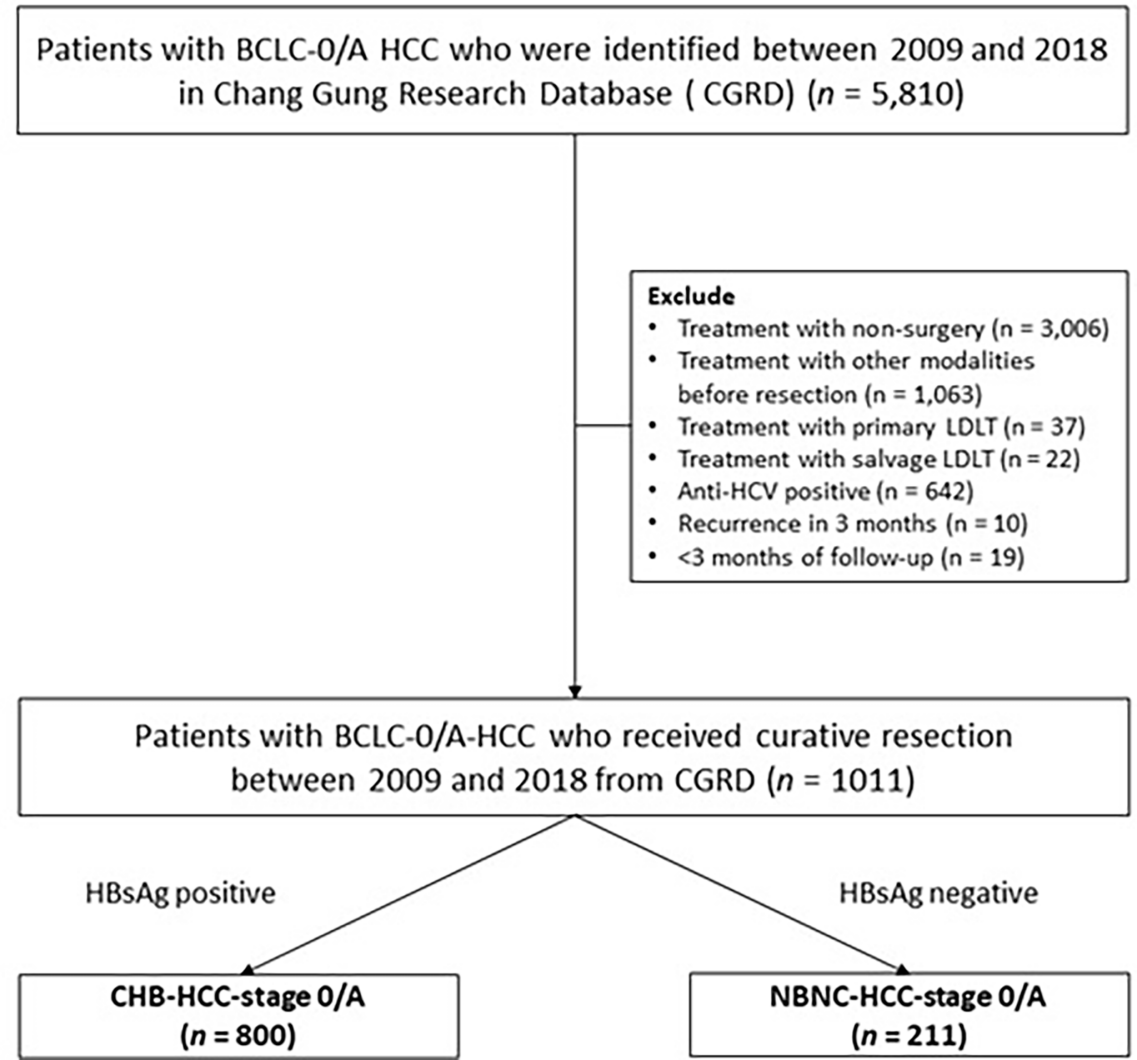
Patient selection flow diagram.

We retrospectively reviewed a total of 5810 patients who were diagnosed with BCLC-0/A HCC between January 2009 and 2018 using the Chang Gung Research Database (CGRD), which is derived from the largest private hospital system in Taiwan; the database is systematically updated annually to include new data generated at CGMH. The CGRD data is obtained from two medical centers and two regional hospitals: Keelung, Linkou, Chiayi, and Kaohsiung CGMH. We excluded 3006 patients who received non-surgical treatment, 1063 patients who underwent prior treatment for HCC, 642 anti-HCV-positive patients, and 10 patients who developed recurrence within less than 3 months after resection. In well-selected patients, liver transplantation is generally considered to cure the tumor and underlying cirrhosis at the same time, and thus strongly influences survival and recurrence (28). Therefore, 59 patients who underwent salvage liver transplantation were also excluded. Finally, after excluding 19 patients who were followed-up for less than three months, a total of 1011 patients with BCLC-0/A-HCC who received curative resection (**Figure 1**) were included in this study; 800 of these patients had CHB-HCC-stage 0/A and the other 211 patients had HCC NBNC-HCC-stage 0/A.

### Assessment and Follow-Up Evaluation

All data were collected retrospectively from medical records at the time of surgery, including age, gender, presence of DM, hypertension, alcohol consumption, smoking history, serum biochemistry, and hepatitis B surface antigen. The histological features of the resected tumor, including satellite nodules, capsule invasion, microvascular invasion, tumor differentiation, histologic grade, and cirrhosis were documented in pathologic reports. The end date of follow-up was 31 December 2018.

### Definitions

NBNC was defined as cases negative for markers of both HBsAg and HCV-Ab (29). Resolved HBV infection was defined as the clearance of HBsAg, with antibodies to hepatitis B core antigen (anti-HBc) and with or without the development of antibodies to HBsAg (anti-HBs) (30). MAFLD was defined as pathologically diagnosed hepatic steatosis and the presence of any one or more of the following three conditions: overweight/obesity, type 2 diabetes mellitus (T2DM), or evidence of metabolic dysregulation. The metabolic dysregulation was defined as the presence of two or more of the following conditions: waist circumference ≥ 90/80 cm in Asian men and women, blood pressure ≥ 130/85 mm Hg or specific drug treatment, plasma triglycerides ≥ 150 mg/dL or specific drug treatment, plasma high-density lipoprotein cholesterol < 40 mg/dL for men and <50 mg/dL for women or specific drug treatment, prediabetes, a homeostasis model assessment of insulin resistance score (HOMA-IR index) ≥ 2.5, or a plasma high-sensitivity C-reactive protein (hs-CRP) level > 2 mg/L (26).

The diagnosis of HCC was defined according the histopathology reports for the surgically resected tumor tissues and defined according to the BCLC guidelines (31). The histologic grade of tumor differentiation was assessed using the Edmondson grading system (32). Liver cirrhosis was defined as an Ishak fibrosis score 5–6 for the non-tumor tissues (33). T2DM was defined based on the World Health Organization (WHO) National diabetic group criteria (34).

### Statistical Analysis

Statistical analyses were performed using SPSS Version 23.0 (IBM Corp., Armonk, NY, USA) for Windows. Continuous variables were expressed as means ± standard deviations, while categorical variables were summarized as frequencies and relative percentages. The Kaplan-Meier method was used to plot the RFS and OS curves for patients stratified by HCC with CHB or NBNC, and the curves were compared using the log-rank test. Factors that were significant in the univariate analysis (*p*< 0.05) were included in multivariate analyses of OS and RFS using a Cox forward stepwise variable selection process. Hazard ratios (HR) and 95% confidence intervals (CI) were also calculated for each factor. *P*-values < 0.05 were considered statistically significant.

## Results

### Patient Characteristics

**Table 1** summarizes the characteristics of the study cohort, which included 852 males and 159 females. Of the 1011 patients, 800 (79.1%) were in the CHB-HCC group and 211 (20.9%) were in the NBNC group. Compared to the CHB-HCC group, the patients in the NBNC group were significantly older (64.2 vs. 56.2 years old, *p*< 0.001) and had a higher body mass index (BMI; 26.2 vs. 25.2%, *p*< 0.001), more frequently had T2DM (51.2 vs. 24.6%, *p*< 0.001), hypertension (55.0 vs. 33.4%, *p*< 0.001) and consumed alcohol (23.3 vs. 11.8%, *p*< 0.001), had lower serum AFP (22.7 vs. 43.8%, *p*< 0.001) and a lower Ishak score (3.5 vs. 4.1,*p*< 0.001), were less likely to have liver cirrhosis (36.0 vs. 50.0%, *p*< 0.001), microvascular invasion (14.7 vs. 30.7%, *p*< 0.001) and capsule invasion (64.0 vs. 80.6%, *p*< 0.001), were more likely to have MAFLD (58.5 vs. 45.6%, *p*< 0.001) and well-differentiated histological grade (*p*< 0.001), and had larger tumors (3.2 vs. 2.7 cm, *p*< 0.001).

**Table 1:** Characteristics of the CHB-HCC and NBNC-HCC group.

	CHB-HCC (n = 800)	NBNC-HCC (n = 211)	P value
Age (years), mean ± SD	56.2± 10.7	64.2± 10.8	<0.001
Male gender, n (%)	683 (85.4)	169 (80.1)	0.061
BMI (kg/m^2^), mean ± SD	25.2± 3.6	26.2± 3.9	0.001
Diabetes mellitus, n (%)	196 (24.6)	108 (51.2)	<0.001
Hypertension, n (%)	267 (33.4)	116 (55.0)	<0.001
Alcohol drinking			<0.001
Never, n (%)	588 (73.6)	142 (67.6)	
Current, n (%)	94 (11.8)	49 (23.3)	
Quit, n (%)	117 (14.6)	19 (9.0)	
Platelet (<150 10^3^/μL), n (%)	299 (38.7)	68 (33.2)	0.144
AST (U/L), mean ± SD	36.8 ± 20.8	41.8 ± 54.7	0.200
ALT (U/L), mean ± SD	42.6 ± 34.6	43.3 ± 63.5	0.827
Total bilirubin (mg/dL), mean ± SD	0.8 ± 0.4	0.8 ± 0.6	0.330
Albumin (g/dL), mean ± SD	4.2 ± 0.5	4.2 ± 0.5	0.404
Creatinine (mg/dL), mean ± SD	1.1 ± 3.0	1.1 ± 1.6	0.382
AFP (>20 ng/mL), n (%)	342 (43.8)	48 (22.7)	<0.001
AFP (>200 ng/mL), n (%)	168 (21.5)	16 (7.6)	<0.001
Child-Pugh (A/B), n (%)	755 (99.0)/ 8 (1.0)	207 (98.1)/ 4 (1.9)	0.323
Ishak score, mean ± SD	4.1 ± 1.9	3.5 ± 2.0	<0.001
Liver cirrhosis, n (%)	400 (50.0)	76 (36.0)	<0.001
BCLC stage 0/A, n (%)	209 (26.1)/ 591 (73.9)	37 (17.1)/ 175 (82.9)	0.006
Tumor size (cm), mean ± SD	2.7 ± 1.0	3.2 ± 1.7	<0.001
Multiple tumors, n (%)	94 (11.8)	22 (10.4)	0.592
Histology grade			<0.001
Well, n (%)	145 (18.2)	50 (23.7)	
Moderate, n (%)	503 (63.1)	131 (62.1)	
Poor, n (%)	149 (18.7)	30 (14.2)	
Microvascular invasion, n (%)	245 (30.7)	31 (14.7)	<0.001
Capsule invasion^*^, n (%)	644 (80.6)	135 (64)	<0.001
Satellite nodule, n (%)	24 (3.0)	6 (2.8)	0.896
MAFLD, n (%)	365 (45.6)	120 (58.5)	0.001
Follow-up (months), mean ± SD	65.6 ± 32.7	65.4 ± 31.4	0.845

Data are expressed as mean ± standard deviation or n (%).

CHB, chronic hepatitis B; NBNC, non-B non-C; BMC, body mass index; AST, aspartate aminotransferase; ALT, alanine aminotransferase; AFP, alpha fetoprotein; MAFLD, metabolic-associated fatty liver disease.

^*^Including partial and totally capsule invasion, and no well capsule.

### Comparison of the 2009–2013 and 2014–2018 Cohorts of Patients With NBNC HCC and HBV HCC

**Table 2** compares the clinicopathologic characteristics of the patients with CHB-HCC and NBNC-HCC in the study cohort from the first five-year period (2009-2013) and the second five-year period (2014-2018).

**Table 2:** Comparison of the clinicopathologic characteristics of the CHB-HCC and NBNC-HCC patients from the first 5 year (2009-2013) to the 2^nd^ 5 year (2014-2018).

	The 1^st^ 5 year (2009-2013)	The 2^nd^ 5 year (2014-2018)	Pvalue	The 1^st^ 5 year (2009-2013)	The 2^nd^ 5 year (2014-2018)	Pvalue
	CHB-HCC (n = 384)	CHB-HCC (n = 416)		NBNC-HCC (n = 80)	NBNC-HCC (n = 131)	
Age (years)	55.9± 10.9	56.5± 10.5	0.423	62.3± 11.6	65.4± 10.1	0.047
Male gender	326 (84.9)	357 (85.8)	0.713	65 (81.3)	104 (79.4)	0.743
BMI (kg/m^2^)	25.0± 3.4	25.4± 3.7	0.173	25.1± 3.7	26.8± 3.9	0.002
DM	91 (23.8)	105 (25.3)	0.613	34 (42.5)	74 (56.5)	0.049
HTN	119 (31.1)	148 (35.6)	0.177	34 (42.5)	82 (62.6)	0.004
Alcohol drinking			0.614			0.082
Never	278 (72.0)	310 (74.5)		55 (68.8)	87 (66.9)	
Current	44 (11.5)	50 (12.0)		22 (27.5)	27 (20.8)	
Quit	61 (15.9)	56 (13.5)		3 (3.8)	16 (12.3)	
MAFLD	175 (45.6)	190 (45.7)	0.977	35 (45.5)	85 (66.4)	0.003
Liver cirrhosis	205 (53.1)	196 (47.1)	0.089	24 (30.0)	52 (39.7)	0.155
Child-Pugh			0.617			0.615
Class A	350 (99.2)	405 (98.8)		38 (97.5)	129 (98.5)	
Class B	3 (0.8)	5 (1.2)		2 (2.5)	2 (1.5)	
Tumor size (cm)	2.8 ± 1.1	2.7 ± 1.0	0.136	3.4 ± 2.2	3.0 ± 1.1	0.134
Tumor size (>2cm)	303 (78.9)	304 (73.1)	0.054	69 (86.3)	113 (86.3)	0.989
BCLC			0.032			0.895
Stage 0	87 (22.7)	122 (29.3)		14 (17.5)	22 (16.8)	
Stage A	297 (77.3)	294 (70.7)		66 (82.5)	109 (83.2)	
AFP (>20 ng/mL)	166 (45.1)	176 (42.7)	0.502	18 (22.5)	30 (22.9)	<0.001
AFP (>200 ng/mL)	79 (21.5)	89 (21.6)	0.964	7 (8.8)	9 (6.9)	<0.001
Histology grade			0.899			0.110
well	72 (18.8)	73 (17.7)		16 (20.0)	34 (26.0)	
moderate	242 (63.0)	261 (63.2)		55 (68.8)	76 (58.0)	
poor	70 (18.2)	79 (19.1)		9 (11.2)	21 (16.0)	
Microvascular invasion	112 (29.2)	133 (32.0)	0.377	9 (11.3)	22 (16.8)	0.001
Capsule invasion	321 (83.6)	323 (77.8)	0.040	48 (60)	87 (66.4)	0.008
Satellite nodule	14 (3.7)	10 (2.4)	0.297	2 (2.5)	4 (3.1)	0.684
Resolved CHB^*^				40 (81.6)	53 (93.0)	0.076

Data are expressed as mean ± standard deviation or n (%).

CHB, chronic hepatitis B; NBNC, non-B non-C; BMI, body mass index; DM, diabetes mellitus; HTN, hypertension; AFP, alpha fetoprotein; MAFLD, metabolic-associated fatty liver disease; LC, liver cirrhosis.

^*^Resolved CHB means patients who are HBsAg negative and anti-HBc positive.

In the second five-year period, patients with CHB-HCC were significantly more likely to have BCLC 0 instead of BCL A (*p* = 0.032) and were less likely to have capsule invasion (*p* = 0.040).

Compared to the cohort from 2009-2013, the patients with NBNC-HCC in 2014-2018 were significantly older (*p* = 0.047) and had a higher BMI (*p* = 0.002) and higher AFP (*p*< 0.001), and more frequently had T2DM (*p* = 0.049), hypertension (*p* = 0.004) and MAFLD (*p* = 0.003).

**Figure 2:**
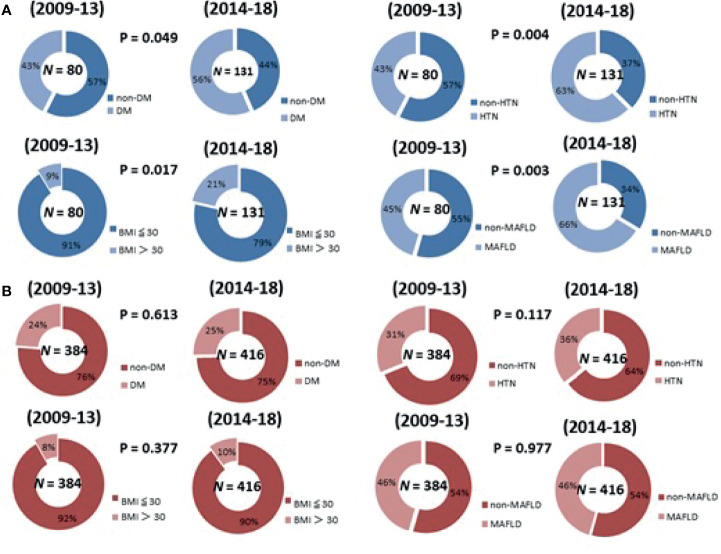
The change of the proportion of metabolic disorders in 2009-2018 in patients with **(A)** NBNC-HCC patients **(B)** CHB-HCC patients.

**Figure 2** shows that, compared to the first five-year period (2009-2013), the patients with NBNC-HCC in the second five-year period (2014-2018) had a higher proportion of metabolic disorders, with significant increases in the frequency of DM (*p* = 0.049), HTN (*p* = 0.004), higher BMI (*p* = 0.017), and MAFLD (*p* = 0.003). However, among the patients with CHB-HCC, there was no significant change in the proportions of patients with metabolic disorders between 2009-2013 and 2014-2018.

### Comparison of the Outcomes of NBNC HCC and HBV-HCC

**Figure 3:**
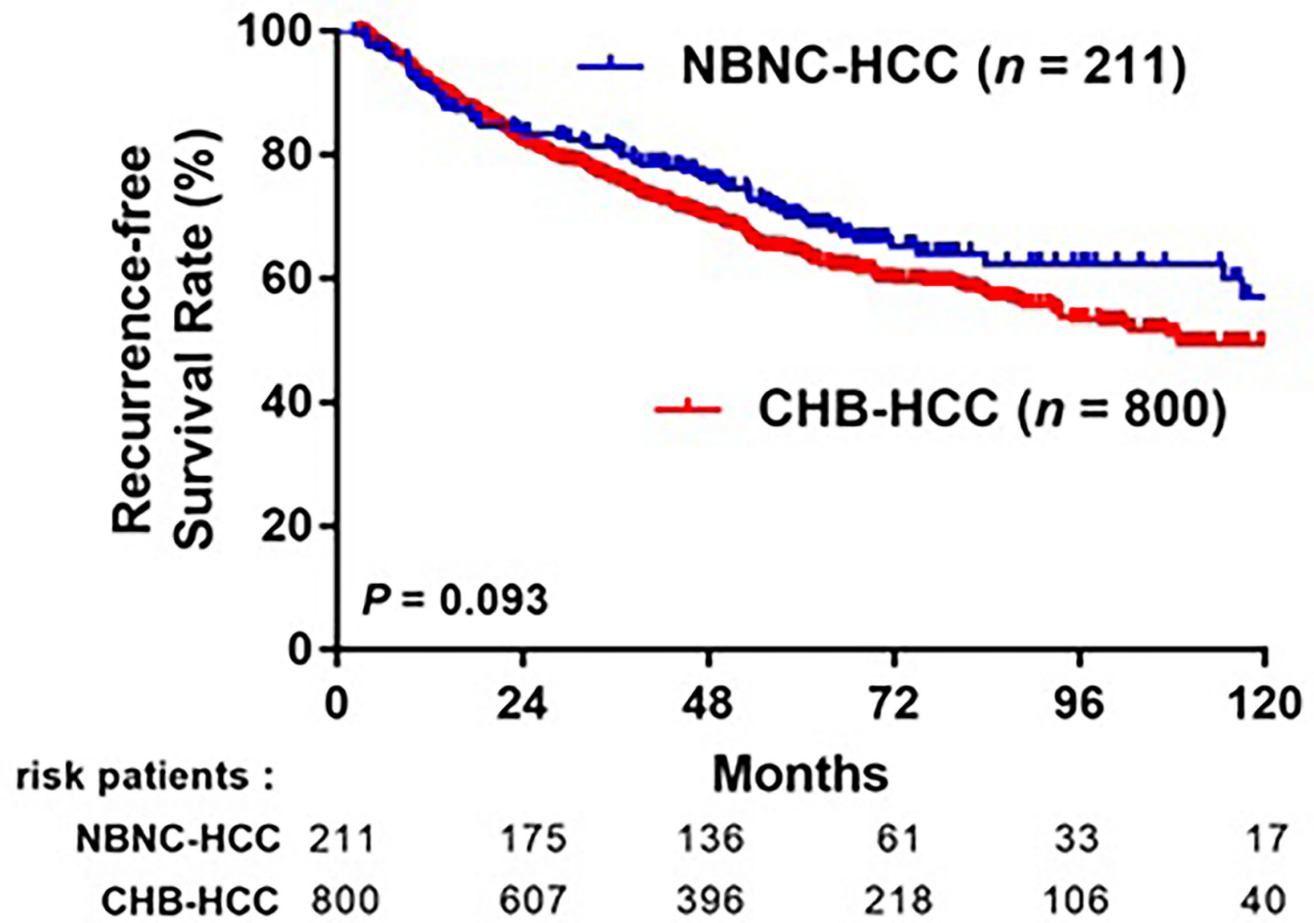
Kaplan-Meier curves of overall recurrence-free survival (RFS) in patients with CHB-HCC or NBNC-HCC receiving curative resection.

**Figure 4:**
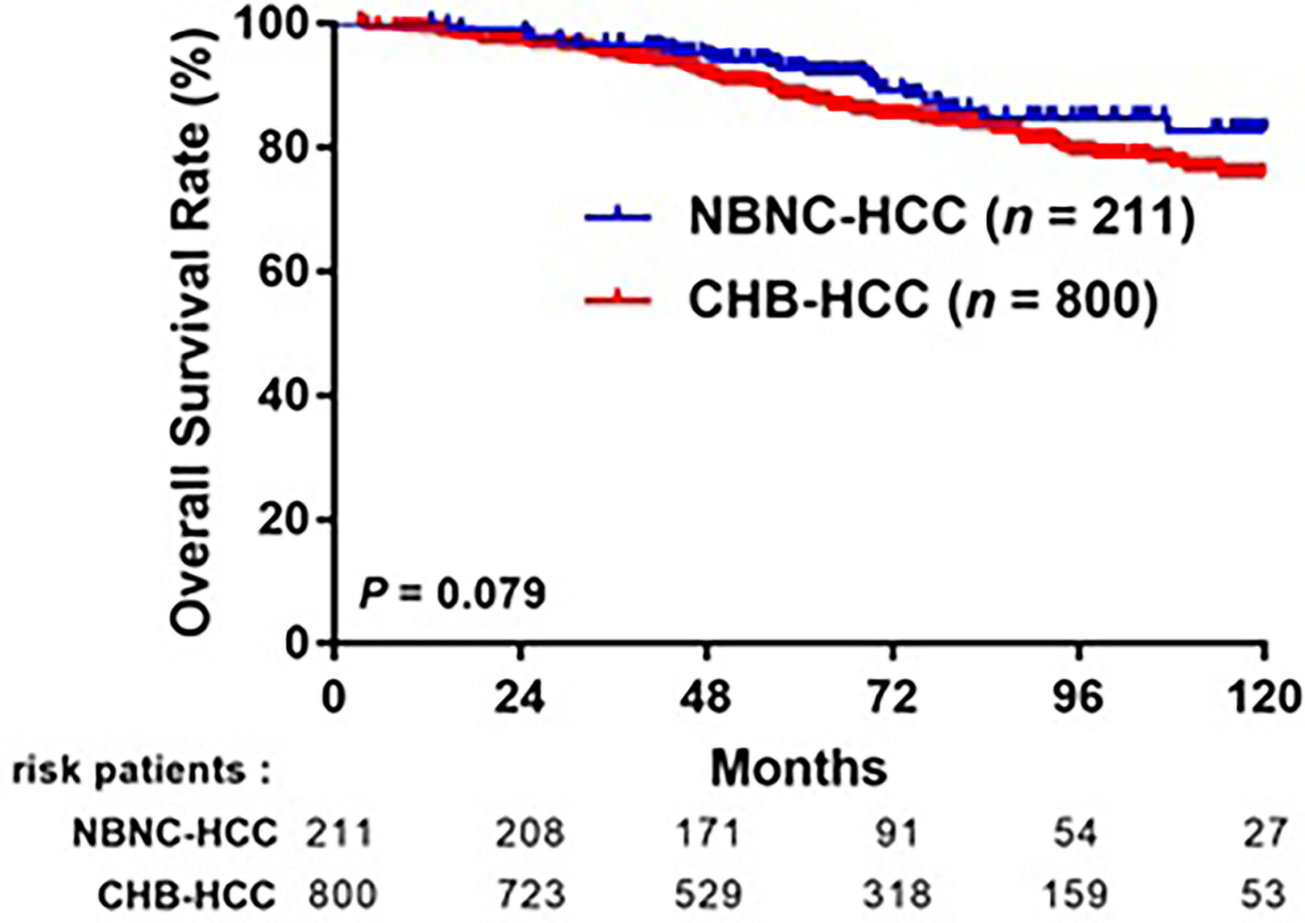
Comparison overall survival (OS) after curative resection between patients with CHB-HCC and NBNC-HCC.

After a median follow-up of 64 months, 363 (36.3%) patients experienced HCC recurrence: 293 (36.6%) in the CHB-HCC group and 70 (33.2%) in the NBNC-HCC group. The 1-, 3-, 5-year RFS rates were 91.4%, 75.8%, and 64.2%, respectively, in the CHB-HCC group; compared to 90.5%, 80.9%, and 70.2%, respectively, in the NBNC-HCC group (*p* = 0.093, **Figure 3**). A total of 134 (13.3%) patients died during the follow-up period: 111 (13.9%) in the CHB-HCC group and 23 (10.9%) in the NBNC-HCC group. The overall survival rates at 1-, 3-, and 5-years were 99.0%, 95.0%, and 88.3%, respectively, in the CHB-HCC group; compared to 100%, 96.7%, and 92.7%, respectively, in the NBNC-HCC group (*p* = 0.079, **Figure 4**). Although there were no statistically significant differences between patients with NBNC HCC and HBC HCC, the patients with NBNC-HCC had more favorable RFS and OS rates than the patients with CHB-HCC.

### Factors Associated With HCC Recurrence

The stepwise Cox proportional hazard model shown in **Table 3** summarizes the prognostic factors associated with HCC recurrence in the entirestudy cohort. In this model, diabetes mellitus (hazard ratio [HR], 1.426; 95% CI, 1.133-1.795, *p* = 0.003), liver cirrhosis (HR, 1.965; 95% CI, 1.571-2.458, *p*< 0.001), a higher tumor number (HR, 1.350; 95% CI, 1.007-1.809, *p* = 0.045), larger tumor size (cm) (HR, 1.108; 95% CI, 1.035-1.187, *p* = 0.003), satellite nodules (HR, 1.910; 95% CI, 1.094-3.333, *p* = 0.023) and CHB (HR, 1.339; 95% CI, 1.010-1.775, *p* = 0.043) were related to a higher risk of recurrence.

**Table 3:** Prognostic factors associated with HCC recurrence.

Variable	Comparison	Univariate		Multivariate	
		HR (95%CI)	P value	HR (95%CI)	P value
Age (years)	Per 1-year increase	1.013 (1.004-1.023)	0.004		
Sex	Male vs Female	1.140 (0.848-1.532)	0.387		
Diabetes mellitus	Yes vs No	1.467 (1.182-1.819)	<0.001	1.426 (1.133-1.795)	0.003
Hypertension	Yes vs No	1.173 (0.950-1.447)	0.138		
Alcohol drinking	Current vs Never/Past	1.018 (0.811-1.278)	0.877		
Smoking	Current vs Never/Past	1.125 (0.907-1.396)	0.283		
HCC family history	Yes vs No	1.263 (0.966-1.652)	0.088		
Platelet (10^9^/L)	<150 vs ≧150	1.467 (1.185-1.815)	<0.001		
AFP (ng/mL)	>10 vs ≦10	1.205 (0.977-1.486)	0.082		
Child-Pugh class	B vs A	0.897 (0.335-2.406)	0.830		
ALBI grade	II/III vs I	1.095 (0.850-1.409)	0.483		
Liver cirrhosis	Yes vs No	1.983 (1.607-2.448)	<0.001	1.965 (1.571-2.458)	<0.001
BCLC stage	A vs 0	1.382 (1.066-1.791)	0.015		
Tumor no.	Multiple vs Single	1.291 (0.966-1.724)	0.084	1.350 (1.007-1.809)	0.045
Tumor size (cm)	Per 1 increase	1.075 (1.011-1.143)	0.020	1.108 (1.035-1.187)	0.003
Histology stages	Poor/Moderate vs Well	1.241 (0.944-1.632)	0.122		
Microvascular invasion	Yes vs No	1.303 (1.041-1.629)	0.021		
Capsule invasion	Yes vs No	1.130 (0.880-1.452)	0.338		
Satellite nodules	Yes vs No	2.314 (1.421-3.768)	0.001	1.910 (1.094-3.333)	0.023
MAFLD	Yes vs No	0.923 (0.750-1.135)	0.445		
Etiology of HCC	CHB vs NBNC	1.250 (0.963-1.624)	0.094	1.339 (1.010-1.775)	0.043

HR, hazard ratio; CI, confidence interval; AFP, alpha fetoprotein; ALBI, albumin-bilirubin; HCC, hepatocellular carcinoma; MAFLD, metabolic associated fatty liver disease.

### Factors Associated With HCC Overall Mortality

As shown in **Table 4**, multivariate analysis revealed that older age (HR: 1.026, 95% CI: 1.007-1.045, *p* = 0.006), presence of cirrhosis (HR: 2.122, 95% CI: 1.424-3.161, *p*< 0.001),lowerplatelets (HR, 1.568; 95% CI, 1.078-2.281, *p* = 0.019), microvascular invasion (HR: 1.459, 95% CI: 1.012-2.103, *p* = 0.043), larger tumor size (HR: 1.496, 95% CI: 1.289-1.736, *p*< 0.001), and CHB (HR, 1.700; 95% CI, 1.017-2.842, *p* = 0.043) were independent risk factors associated with death.

**Table 4:** Prognostic factors associated with overall mortality.

Variable	Comparison	Univariate		Multivariate	
		HR (95%CI)	P value	HR (95%CI)	P value
Age (years)	Per 1-year increase	1.020 (1.004-1.035)	0.012	1.026 (1.007-1.045)	0.006
Sex	Male vs Female	1.163 (0.716-1.890)	0.542		
Diabetes mellitus	Yes vs No	1.127 (0.782-1.624)	0.522		
Hypertension	Yes vs No	1.181(0.835-1.669)	0.347		
Alcohol drinking	Current vs Never/Past	1.207 (0.884-1.815)	0.197		
HCC family history	Yes vs No	0.803 (0.489-1.320)	0.387		
Platelet (10^9^/L)	<150 vs ≧150	1.971 (1.386-2.803)	<0.001	1.568 (1.078-2.281)	0.019
AFP (ng/mL)	>10 vs ≦10	1.075 (0.763-1.514)	0.679		
Child-Pugh class	B vs A	2.844 (1.050-7.705)	0.040		
ALBI grade	II/III vs I	1.337 (0.901-1.986)	0.149		
Liver cirrhosis	Yes vs No	2.278 (1.593-3.256)	<0.001	2.122 (1.424-3.161)	<0.001
BCLC stage	A vs 0	1.538 (0.973-2.429)	0.065		
Tumor no.	Multiple vs Single	1.061 (0.652-1.725)	0.813		
Tumor size (cm)	Per 1 increase	1.124 (1.039-1.217)	0.004	1.496 (1.289-1.736)	<0.001
Histology stages	Poor/Moderate vs Well	1.100 (0.707-1.711)	0.672		
Microvascular invasion	Yes vs No	1.596 (1.125-2.263)	0.009	1.459 (1.012-2.103)	0.043
Capsule invasion	Yes vs No	1.397 (0.899-2.173)	0.138		
Satellite nodules	Yes vs No	2.591 (1.267-5.298)	0.009		
MAFLD	Yes vs No	0.737 (0.523-1.039)	0.081		
Etiology of HCC	CHB vs NBNC	1.492 (0.952-2.340)	0.081	1.700 (1.017-2.842)	0.043

HR, hazard ratio; CI, confidence interval; AFP, alpha fetoprotein; ALBI, albumin-bilirubin; HCC, hepatocellular carcinoma; MAFLD, metabolic associated fatty liver disease.

## Discussion

This study aimed to evaluate the changesin the frequency of metabolic disorders in patients with NBNC-HCC and CHB-HCC in Taiwan during the period from 2009-2018. In this large multicenter study, we retrospectively assessed the clinical characteristics of 1011 patients who were classified into the NBNC-HCC and CHB-HCC groups after curative resection for early-stage HCC (BCLC stage 0 or A). The main finding was that the the frequency of metabolic dysregulation, including DM, HTN, BMI > 30, and MAFLD, tended to increase over time among patients with NBNC-HCC, but not among patients with HBV-HCC. To our knowledge, this is the first study to apply the new concept of MAFLD in a comparison of patients with NBNC-HCC and CHB-HCC. Moreover, the patients with HBV-HCC had a poorer prognosis, which was associated with more advanced pathological features, compared to the patients with NBNC-HCC.

The increasing incidence of metabolic diseases worldwide and the rising prevalence of MAFLD make their coexistence with other chronic liver diseases highly possible. An international panel of experts proposed the new concept of MAFLD in 2020 (26, 27, 35). The prevalence of NAFLD is growing dramatically; however, this term lacks clear nomenclature for nonalcohol-use-disorder fatty liver disease, and has an absence of properly defined “positive” diagnostic criteria. The new definition of MAFLD places increased emphasis on the important role of metabolic dysfunction in liver disease (36, 37). A recent cohort study by Huang et al. classified patients with histology-proven hepatic steatosis and cryptogenic cirrhosis according to the diagnostic criteria for MAFLD and NAFLD, and compared their clinical and histologic features (38). They confirmed that the novel diagnostic criteria for MAFLD exhibited a higher degree of disease severity in terms of histologic and laboratory data and helped to better identify patientsthan the criteria for NAFLD. Wang et al. reported that patients with HBV-MAFLD had similar metabolic features as patients with MAFLD alone in a cohort study in China (39). However, NBNC-HCC, which is considered to be related to metabolic disorders, has not been studied using the new MAFLD criteria. Moreover, it is worth noting that the proportions of patients diagnosed various types of HCC with this new standard still remain unclear.

In this study, we applied the concept of MAFLD to a comparison of patients with CHB-HCC and NBNC-HCC over two five-year periods. There was no difference in the ratio of MAFLD to CHB between 2009-13 and 2014-18. However, the proportions of patients with NBNC-HCC increased from 45.5% in the first five-year period (2009-13) to 66.4% (*p* = 0.003) in the second five-year period (2014-18; *p* = 0.003). A large population cohort conducted by Myers et al. revealed that the burden of NAFLD- and MAFLD-associated HCC increased significantly between 1990and 2014, and this trend accounted for an increase in the incidence of HCC (40). However, as shown in **Figure 2**, the incidence of MAFLD and metabolic syndrome did not increase among patients with CHB-HCC in our study. This result provides evidence that the increased incidence of MAFLD in HCC is largely due to a higher proportion of MAFLD in patients with NBNC-HCC. Moreover, when we further investigated the assessment criteria for MAFLD, the patients with NBNC-HCC in the second five-year period were significantly older, and had a higher frequency of T2DM, hypertension, and higher BMI compared to the patients with HBV-HCC. This result is similar, but not exactly the same, as recently published Japanese study (41). Nagaoki et al. demonstrated that the numbers of patients with NBNC-HCC with metabolic syndrome (T2DM, hypertension, hyperlipidemia) increased significantly between 1992–2009 and 2010–2018. However, we found that patients with NBNC-HCC had a high frequency of metabolic disorders over the 10 years from 2009 and 2018. Moreover, Nagaoki et al. compared the overall incidence and the number of patients with NBNC-HCC, whereas we focused on patients with BCLC-0/A-HCC who received resection, which remains the mainstay curative treatment. Although it is still unclear whether metabolic disorders affect HCC recurrence among patients with NBNC, this result can possibly be used as a basis for further studies. And for NBNC-HCC, the increase in metabolic syndrome may be related to the change of diet and health in Taiwan. Wen-Harn et al. uses 24 hour dietary recall data from the 1993-1996 and 2005-2008 to assessed the trends in dietary habits. They found the dietary habits in Taiwan are changing which has led to the increase in obesity and associated metabolic diseases (42). Many previous study has confirmed that metabolic syndrom and fatty liver is highly correlated with the occurrence of HCC (22, 43). A review conducted by Daniel Q et al. explained how metabolic syndrome exacerbates the process of NAFLD/NASH leading to the development of HCC (44). There are two major causes of metabolic syndrome leading to HCC, including inducing apoptosis and small intestinal bacterial overgrowth (SIBO). In obesity, excessive fat accumulation results in overexpansion of adipose tissue, resulting in adipocyte cell death which promotes stimulate Inflammasomes, cytokines (TNF-α, IL-6 and iNOS) and hepatic stellate cells. Release of inflammatory cytokines from adipose tissue further contributes to liver inflammation, NAFLD/NASH and higher incidence of liver cancer (45). Another reason that cannot be ignored is that SIBO altered intestinal permeability may facilitate the passage of bacteria derived products into the systemic circulation, causing a systemic inflammatory state and destruction of the intestinal mucosa barrier (46). Although the detailed mechanism is still unclear, an experiment showed that the intestinal mucosal barrier function has changed, leading to the progression of NAFLD in rats (47). Taken together, based on this study, we postulate that high proportions of patients with NBNC-HCC will tend to have MAFLD, obesity, T2DM, and hypertension in the future. Therefore, monitoring metabolic syndrome and MAFLD among patients with NBNC-HCC after curative resection will be crucial; however, additional studies are needed to confirm this hypothesis.

We further compared the characteristics of the CHB-HCC and NBNC-HCC groups. The patients with HBV-HCC were younger, less frequently had T2DM or hypertension, had a lower BMI, and were more likely to have liver cirrhosis, capsule invasion, and vascular invasion. The results of a Japanese study support our findings (48): Xue et al. confirmed that patients with HBV-HCC were younger and more likely to have vascular invasion compared to patients with NBNC-HCC. Additionally, these risk factors—including liver cirrhosis, capsule invasion, and vascular invasion—may be associated with certain carcinogenic features of HBV, such as increased cell motility caused by HBxAg (49, 50). Therefore, we further investigated the outcomes of the patients with CHB-HCC and NBNC-HCC. Despite the fact that the differences in RFS and OS were only close to statistically significant, NBNC was considered to be a better prognostic factor than HCV-HCC in multivariate analysis, in agreement with other previous studies. Okuda et al. retrospectively reviewed 201 patients with HCC who underwent initial hepatectomy, and reported that patients with NBNC had a significantly better five-year survival rate than patients with hepatitis B or C (51). Another comparative study of 11,950 patients indicated that patients with NBNC-HCC had a lower risk of HCC recurrence than patients with HBV-HCC or HCV-HCC (52). Although the detailed underlying mechanisms remain unclear or controversial, the better prognosis of patients with NBNC may be possibly explained by their negative viral status. Similar pathological differences were also noted in the study by Li et al. (53), in which relative to the viral groups, patients with NBNC less frequently had multicentric tumors; this feature has been related to an increased risk of recurrence in HCC (54–56). Hence, we consider that viral infection leads to poorer outcomes by leading to more advanced clinicopathological features; however, further experimental studies are needed to explore the underlying mechanisms.

Another important issue in NBNC-HCC is so-called resolved HBV infection, especially in Taiwan, an HBV-endemic country. In the present study, the proportion of patients with resolved HBV infection was 87.7% (93/106). The proportion of resolved HBV infection did not change significantly between the first five-year period and second five-year period (81.6% to 93%, *p* = 0.076). However, HBcAb is not routinely tested in daily practice at our center, and data were not available for 105 patients (50%). Even so, the fact that metabolic risks increased in the patients with NBNC-HCC over time does not affect the comparison of the risk of resolved HBV infection.

There are several strengths to this research. First, a large sample of patients who recently received treatment (from 2010 to 2019) was collected, thus the baseline characteristics and prevalence of metabolic diseases are close to those of the current population. Second, we only recruited patients diagnosed with BCLC-0/A HCC who underwent curative resection, which provides a more accurate and validated evaluation of recurrence-free survival. Most importantly, we evaluated hepatic steatosis based on the gold standard ‘pathological assessment’ of resected non-tumor tissues, which is more accurate than analyses based on core biopsies or imaging (38).

There are some inherent potential limitations to our study. Firstly, the information and data were retrospectively collected from medical records. Therefore, some important data was lost or insufficient, such as the HOMA-IR index, lipid profiles, or waist circumference. Second, we only enrolled patients from Taiwan, which is an HBV-endemic area. Additional studies are necessary to confirm whether these trends also occur in other cohorts from Asia and in Western countries. Thirdly, occult HBV infection (OBI), defined as HBsAg-negative and HBV DNA-positive, could not be evaluated in the present study because HBV DNA is not routinely measured in HBsAg-seronegative patients with HCC. A study from Hong Kong, another HBV-endemic area, indicated that OBI was detected in around 70% of HBsAg-seronegative patients with HCC (23). OBI should also be considered in this special populationas HBV infection is also endemic in Taiwan. In the future, comprehensive analysis including anti-HBc, hepatitis B surface antibody, and HBV DNA status is required to clarify the role of OBI in these special populations. Finally, the gene polymorphisms including TM6SF2/PNPLA3/MBOAT7 which are highly related to the abnormal metabolic disease and fatty liver disease are not evaluated in our study (57, 58). Futher studies are needed to investigated whether the proportion of SNPs in NBNC-HCC is getting higher.

## Conclusions

In the future, patients with early-stage NBNC may tend to have a higher frequency of MAFLD, obesity, T2DM, and hypertension; thus, metabolic syndrome should be closely monitored in this special population. Moreover, the prognosis of NBNC-HCC is significantly better than that of CHB-HCC, which may be related to the quite distinct clinicopathological features of these types of HCC.

## Data Availability Statement

The original contributions presented in the study are included in the article/supplementary material. Further inquiries can be directed to the corresponding author.

## Ethics Statement

The institutional review board of Kaohsiung Chang Gung Memorial Hospital approved this study (IRB: 202001658B0 CMRPG8L0261). Written informed consent for participation was not required for this study in accordance with the national legislation and the institutional requirements.

## Author Contributions

Conception and design: M-CT. Manuscript writing: Y-PL. Collection and assembly of data: Y-PL, C-HC, C-CY, Y-WL, P-YH. Data analysis and interpretation: C-CY, M-CT, C-HC, C-CY. All authors contributed to the article and approved the submitted version.

## Conflict of Interest

The authors declare that the research was conducted in the absence of any commercial or financial relationships that could be construed as a potential conflict of interest.

## Publisher’s Note

All claims expressed in this article are solely those of the authors and do not necessarily represent those of their affiliated organizations, or those of the publisher, the editors and the reviewers. Any product that may be evaluated in this article, or claim that may be made by its manufacturer, is not guaranteed or endorsed by the publisher.
